# Cellular Gene Modulation of HIV-Infected CD4 T Cells in Response to Serial Treatment with the Histone Deacetylase Inhibitor Vorinostat

**DOI:** 10.1128/JVI.00351-20

**Published:** 2020-06-16

**Authors:** Jill W. Maxwell, Shane D. Falcinelli, Alexey Nefedov, Corin Dorfmeier, Guoxin Wu, Morgan Dewey, Andrea L. Webber, Nancie M. Archin, David M. Margolis, Daria J. Hazuda, Richard J. O. Barnard, Bonnie J. Howell

**Affiliations:** a Merck & Co., Inc., West Point, Pennsylvania, USA; b UNC HIV Cure Center, University of North Carolina, Chapel Hill, North Carolina, USA; c Department of Microbiology and Immunology, University of North Carolina, Chapel Hill, North Carolina, USA; d Department of Medicine, University of North Carolina, Chapel Hill, North Carolina, USA; Icahn School of Medicine at Mount Sinai

**Keywords:** vorinostat, biomarker, gene expression, histone deacetylase inhibitors, human immunodeficiency virus

## Abstract

Histone deacetylase inhibitors (HDACi) are the most widely studied HIV latency-reversing agents (LRAs). The HDACi suberoylanilide hydroxamic acid (vorinostat [VOR]) has been employed in several clinical HIV latency reversal studies, as well as *in vitro* models of HIV latency, and has been shown to effectively induce HIV RNA and protein expression. Despite these findings, response to HDACi can vary, particularly with intermittent dosing, and information is lacking on the relationship between the host transcriptional response and HIV latency reversal. Here, we report on global gene expression responses to VOR and examine the longevity of the transcriptional response in various cellular models. We found that many genes are modulated at 6 h post-VOR treatment in HCT116, Jurkat, and primary resting CD4 T cells, yet return to baseline levels after an 18-h VOR-free period. With repeat exposure to VOR in resting CD4 T cells, we found similar and consistent transcriptional changes at 6 h following each serial treatment. In addition, serial exposure in HIV-infected suppressed donor CD4 T cells showed consistent transcriptional changes after each exposure to VOR. We identified five host genes that were strongly and consistently modulated following histone deacetylase (HDAC) inhibition; three (H1F0, IRGM, and WIPI49) were upregulated, and two (PHF15 and PRDM10) were downregulated. These genes demonstrated consistent modulation in peripheral blood mononuclear cell (PBMC) samples from HIV-positive (HIV^+^) participants who received either single or multiple doses of 400 mg of VOR. Interestingly, the host transcriptional response did not predict induction of cell-associated HIV RNA, suggesting that other cellular factors play key roles in HIV latency reversal *in vivo* despite robust HDACi pharmacological activity.

**IMPORTANCE** Histone deacetylase inhibitors are widely studied HIV latency-reversing agents (LRAs). VOR, an HDACi, induces histone acetylation and chromatin remodeling and modulates host and HIV gene expression. However, the relationship between these events is poorly defined, and clinical studies suggest diminished HIV reactivation in resting CD4 T cells with daily exposure to VOR. Our study provides evidence that VOR induces a consistent level of host cell gene transcription following intermittent exposure. In addition, in response to VOR exposure a gene signature that was conserved across single and serial exposures both *in vitro* and *in vivo* was identified, indicating that VOR can consistently and reproducibly modulate transcriptional host responses. However, as the HIV response to HDACi declines over time, other factors modulate viral reactivation *in vivo* despite robust HDAC activity. The identified host gene VOR biomarkers can be used for monitoring the pharmacodynamic activity of HDAC inhibitors.

## Abstract (precis)

Histone deacetylase inhibitors are widely studied HIV latency-reversing agents (LRAs). VOR, an HDACi, induces histone acetylation and chromatin remodeling and modulates host and HIV gene expression. However, the relationship between these events is poorly defined, and clinical studies suggest diminished HIV reactivation in resting CD4 T cells with daily exposure to VOR. Our study provides evidence that VOR induces a consistent level of host cell gene transcription following intermittent exposure. In addition, in response to VOR exposure a gene signature that was conserved across single and serial exposures both *in vitro* and *in vivo* was identified, indicating that VOR can consistently and reproducibly modulate transcriptional host responses. However, as the HIV response to HDACi declines over time, other factors modulate viral reactivation *in vivo* despite robust HDAC activity. The identified host gene VOR biomarkers can be used for monitoring the pharmacodynamic activity of HDAC inhibitors.

## INTRODUCTION

Globally, over 39 million people are estimated to be living with HIV, with ∼1.8 million new infections and ∼940,000 deaths each year due to HIV (https://www.who.int/hiv/data/en/). Intensive research over the last three decades has resulted in the development of several antiretroviral agents that inhibit multiple stages of the HIV life cycle. Combined antiretroviral therapy (cART) reduces HIV viremia to below the level of detection of standard viral load assays and has resulted in improved patient health and life span. However, cART is not curative, and cessation invariably leads to viral rebound; it thus requires adherence to lifelong therapy. Strategies aimed at eradicating chronic HIV-1 are a major focus of current research efforts.

One of the barriers to HIV eradication is the persistence of latently infected resting CD4 T cells containing replication competent but transcriptionally silent HIV-1 proviral DNA (1). One strategy for eradication of the HIV reservoir is to use small-molecule HIV latency reversal agents (LRAs) paired with an immune augmentation strategy to deplete the reservoir (2). This approach aims to purge the silent HIV reservoir by using LRAs to induce viral gene transcription and subsequently elicit cell death through augmented immune recognition and killing of infected cells.

Treatment with histone deacetylase inhibitors (HDACi) has emerged as a leading approach for latency reversal, as the compounds induce HIV gene expression without impacting T-cell activation (3, 4). Inhibition of HDACs results in hyperacetylation of histone complexes, leading to chromatin decondensation, increased accessibility of the HIV-1 promoter to the transcriptional machinery, and viral reactivation (4–11). As HDACi also impact acetylation of nonhistone proteins (12), other mechanisms, including the release of free positive transcription elongation factor b (P-TEFb), may also play a key role in HDACi-mediated latency reversal (13).

Proof-of-concept clinical studies using the HDACi suberoylanilide hydroxamic acid (vorinostat [VOR]) (14–16), panobinostat (17), and romidepsin (18) have demonstrated statistically significant increases in HIV RNA in resting CD4 T cells (14–18), cell-associated viral protein (19), and plasma HIV viremia (17, 18) following drug administration. Archin and colleagues were the first to demonstrate the ability of VOR to induce increases in cell-associated HIV RNA (ca-RNA) *in vivo* in resting CD4 T cells after a single 400-mg dose of VOR (14). In a subsequent study, the same group assessed HIV ca-RNA in resting cells after several cycles of 3 daily doses of VOR followed by 4 days of rest (15). This multiple every 24 h (q24h) dosing resulted in minimal increases in HIV ca-RNA relative to the responses observed previously after a single dose of VOR. A more recent study with q72h dosing resulted in a more efficient induction of HIV ca-RNA in resting CD4 T cells across participants relative to q24h dosing, both *ex vivo* and *in vivo* (20). Similarly, intermittent dosing of panobinostat three times a week, followed by a 1-week rest for 8 weeks, resulted in a sustained increase in ca-RNA in total CD4 T cells (17). In contrast, Lewin and colleagues conducted a clinical study in which participants received q24h doses of VOR for 14 days (16). Notably, they observed increased ca-RNA in total CD4 cells from 18 out of 20 HIV-positive (HIV^+^) participants following daily VOR administration for 14 days; this increase was sustained up to 70 days postdosing. However, it is difficult to directly compare these studies, as resting CD4 T cells may differ from total CD4 T cells in their responsiveness to HDACi. Taken together, however, these studies suggest that the dosing interval for HDACi is an important variable for HIV latency reversal, and that in resting CD4 T cells, where most of the long-lived HIV reservoir is found, a refractory period exists in which HIV ca-RNA cannot be increased by VOR dosing. Thus, further study of the temporal host responses (i.e., histone acetylation and transcriptional responses) to serial VOR exposures and their relationship to HIV reactivation may provide important insight into the use of VOR and other HDACi as LRAs in HIV cure strategies.

In clinical studies, dosing of HDACi resulted in measurable increases in histone acetylation, a commonly used proximal biomarker of histone deacetylase (HDAC) target engagement, regardless of dosing interval (14, 15, 17, 18). However, increases in HIV transcription were more variable, indicating that while histone acetylation is a useful measurement for HDACi activity within cells, increases are not predictive of HIV-1 ca-RNA transcriptional response, possibly due to a requirement for additional factor(s) downstream of chromatin decondensation. Host gene biomarkers for HDACi have been explored in the literature (16, 21–23); however, to date no study has validated a set of genes that can be used across multiple cell models and in clinical samples. Furthermore, the relationship between these host gene changes and HIV reactivation has not been thoroughly investigated.

To address these questions, we evaluated host cell transcriptional changes using clinically achievable exposures of VOR in multiple cell types. This allowed identification of a series of candidate mRNA biomarkers for analysis of the stability of host cell transcriptional responses to repeated VOR exposures. Using these biomarkers, we then studied host gene modulation *in vitro* in resting CD4 T cells from healthy donors during serial daily incubations with clinically relevant exposures of VOR. Additionally, these candidate biomarker genes were evaluated in HIV-infected suppressed donor CD4 T cells to evaluate VOR-mediated host cell responses. Finally, using the VOR RNA biomarkers, we investigated the relationship between host cell responses and HIV transcription in participants who received single or multiple daily doses of VOR (15, 20). These studies revealed that in participant-derived samples, host gene transcription, but not that of HIV, consistently responded to VOR treatment. This suggests that while cellular genes respond consistently to VOR *in vivo*, reversal of HIV transcriptional latency is more complex.

Of note, VOR mRNA biomarkers that were identified and validated *in vivo* in our study can be employed in future clinical studies to verify target engagement and assess the pharmacodynamic response to VOR or other HDACi. Gene biomarkers provide an advantage over assessment of histone acetylation, as they may be performed alongside assays for HIV ca-RNA induction and are less cumbersome than measurements of histone acetylation.

## RESULTS

### Identification of HDACi-responsive host genes.

**FIG 1:**
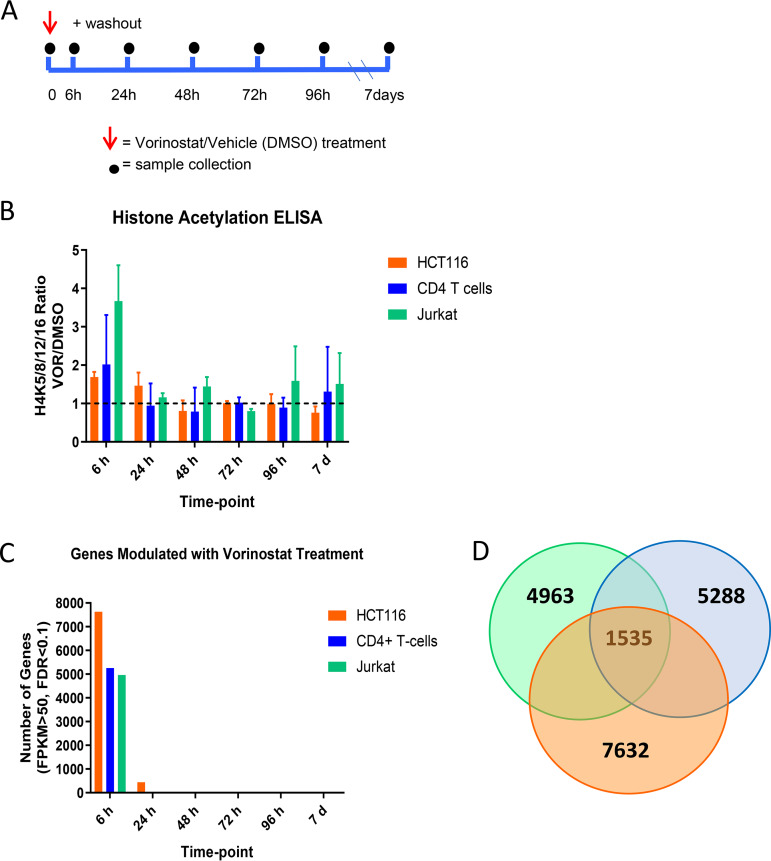
(A) Time course evaluation of 6-h 340 nM VOR treatment over 7 days in HCT116 cells, resting CD4 T cells, and Jurkat cells. (B) Histone acetylation, as measured by enzyme-limited immunosorbent assay (ELISA), showed an increase after 6-h VOR treatment and returned to baseline levels by 24 h. (C) Transcriptome sequencing (RNA-Seq) analysis of genome transcriptional changes measured over 7 days. (D) Venn diagram showing the number of genes distinctly modulated in each cell type and the number shared across cell types.

Histone deacetylase (HDAC) enzymes regulate chromatin structure and gene expression, yet limited data exist on the kinetics of gene expression over time in response to episodic HDAC inhibition. Towards this goal, we examined host gene expression patterns in three cellular models prior to and after treatment with a physiologically relevant dose of 340 nM VOR. Jurkat HIV-1 T cells (a cellular model of HIV latency), HCT116 cells (a cancer cell line used during development of VOR), or uninfected primary resting CD4 T cells were pulsed with 340 nM VOR for 6 h. Cells were subsequently washed to remove VOR and sampled over the course of 7 days (Fig. 1A). Transcriptome sequencing (RNA-Seq) was performed to evaluate host transcriptomic changes, and histone acetylation was used as a pharmacodynamic biomarker for VOR treatment.

Increases in histone acetylation were observed in all cell types following a 6-h VOR treatment and ranged from 1.7 to 3.5-fold relative to that of the vehicle (dimethyl sulfoxide [DMSO]) (Fig. 1B). Histone acetylation levels returned to baseline by 24 h after VOR treatment and remained unchanged until the end of the study.

For evaluating pharmacological biomarkers of HDACi, we employed RNA-Seq and observed modulation of 4963 (Jurkat), 5288 (CD4 T cells), and 7632 (HCT116) genes, following exposure to VOR, using a low stringency cutoff (Fig. 1C; see Data Set S1 in the supplemental material). Analysis of the mRNA expression patterns revealed that 1,535 genes were common to all three cell types (Fig. 1D and the supplemental material), allowing assessment of global transcriptional changes in response to VOR treatment. In order to evaluate a VOR mRNA expression signature, we leveraged these data and similar studies of proximal biomarkers of VOR from the literature (22) and during clinical development of VOR (data not shown; Merck & Co, Inc., Kenilworth, NJ). From these studies, six upregulated and six downregulated candidate biomarker genes (Table 1) were selected based on fold changes and TaqMan probe availability.

**TABLE 1:** Twelve genes that were modified by VOR, identified in previous studies for discovery of proximal biomarkers of HDACi

Gene^*a*^	TaqMan assay ID^*b*^
H1F0 ↑	Hs00271174_s1
IRGM ↑	Hs01013699_s1
CTNNAL1 ↑	Hs00972098_m1
MGC15476 ↑	Hs00376818_m1
WIPI49 ↑	Hs00215872_m1
MAK ↑	Hs00195446_m1
DPEP2 ↓	Hs00902586_m1
PHF15 ↓	Hs00959516_m1
SELL ↓	Hs00174151_m1
PRDM10 ↓	Hs00999748_m1
TNFRSF17 ↓	Hs03045080_m1
KIAA1553 ↓	Hs01028508_s1

a Six genes were upregulated and six were downregulated.

b Used for qPCR validation. ID, identifier.

**FIG 2:**
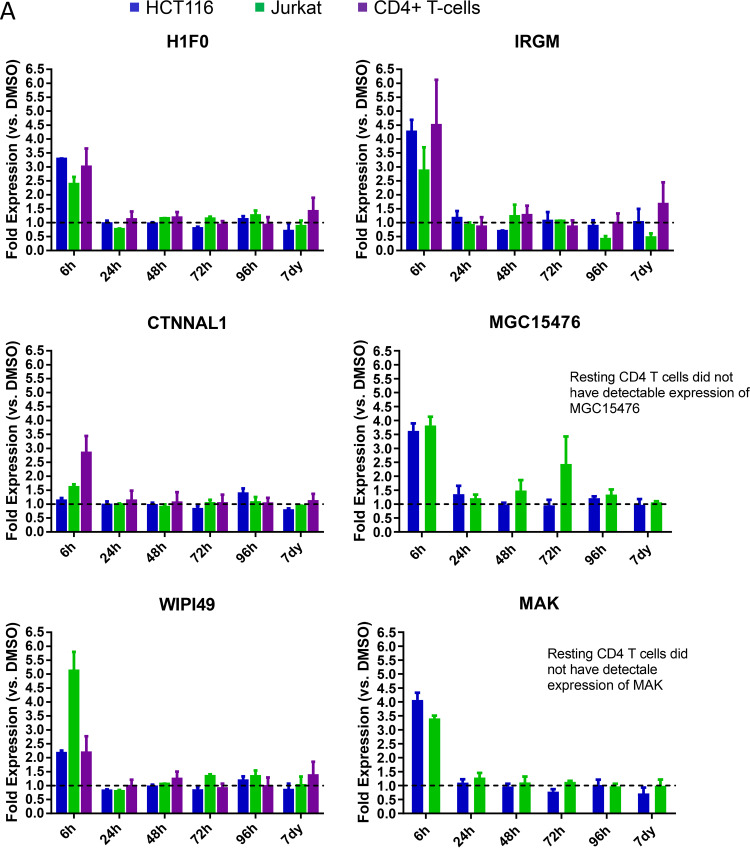

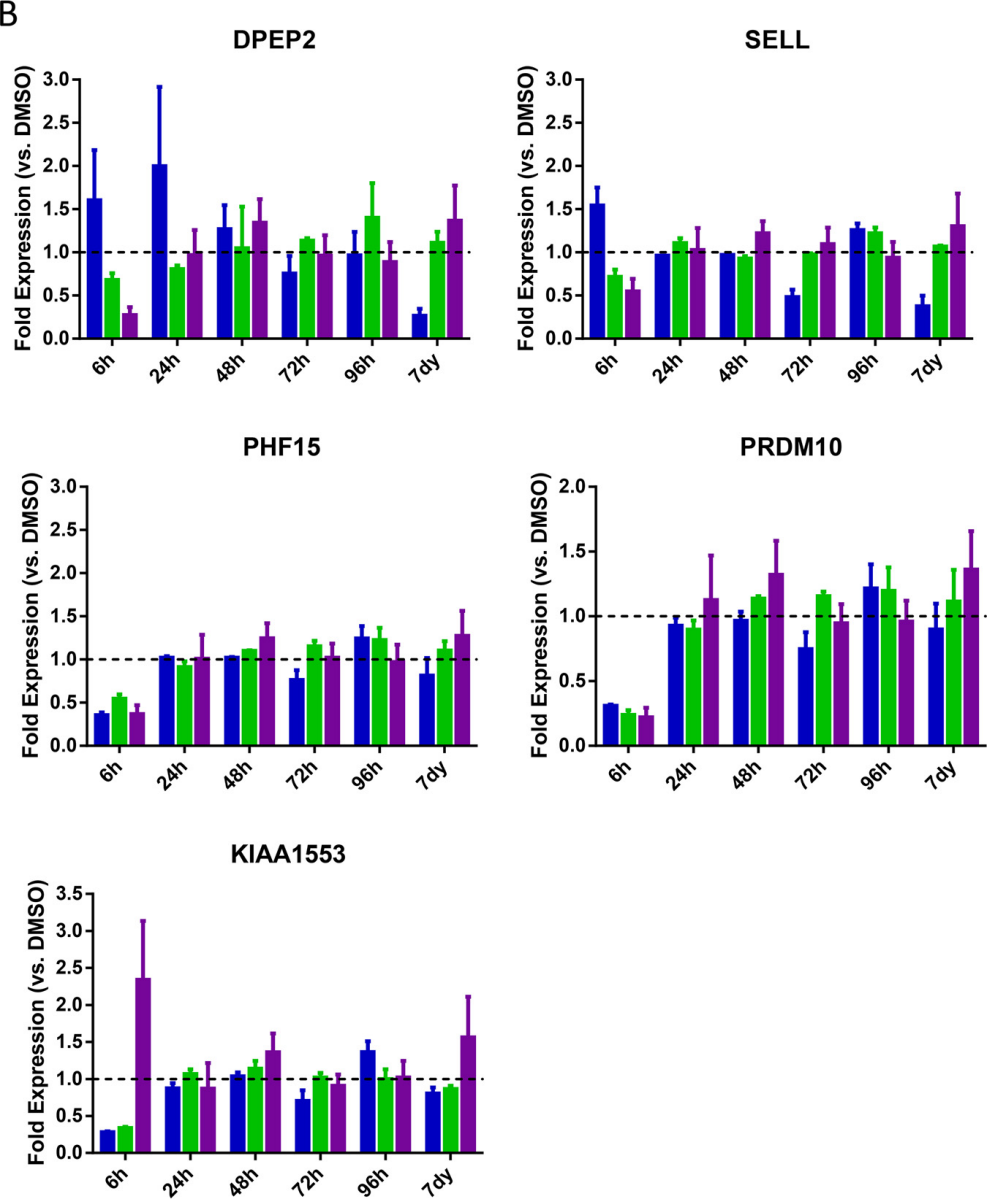
Quantitative PCR validation (fold change) of 12 genes that were identified in previous studies for discovery of proximal biomarkers of HDACi. Analysis of six upregulated (A) and six downregulated (B) genes in Jurkat cells (*n* = 4), CD4 resting T cells (*n* = 4), or HCT116 cells (*n* = 4) treated with a single 6-h 340 nM VOR treatment. Error bars represent standard error of the mean. Five genes show consistent modulation across all three cell types: H1F0, IRGM, WIPI49, PHF15, and PRDM10. Of note, TNFRSF17 did not show detectable expression in any cell type.

We next sought to validate the responsiveness of these genes to VOR in HCT116, Jurkat, and resting CD4 T cells following a single dose of VOR (6-h pulse) by quantitative PCR (qPCR). Of these genes, 5/12 were consistently upregulated (H1F0, IRGM, and WIPI49) or downregulated (PHF15 and PRDM10) across Jurkat, HCT116, and CD4 T cells (Fig. 2). H1F0, IRGM, and WIPI49 showed a 2.5- to 3.5-fold, 3- to 4.5-fold, and 2- to 5-fold increase in expression, respectively, at 6 h after VOR exposure compared to that in DMSO controls (Fig. 2A). In contrast, PHF15 and PRDM10 exhibited a 50 to 70% and 70 to 80% decrease in gene expression at 6 h postdose, respectively (Fig. 2B). Expression of all five genes returned to baseline levels at 24 h and remained at this level throughout the 7-day study in all cell types. Additionally, these five genes also showed response to other HDACi, including panobinostat (data not shown).

### Histones and host genes “respond and reset” with single and serial q24h HDACi exposure *in vitro*.

**FIG 3:**
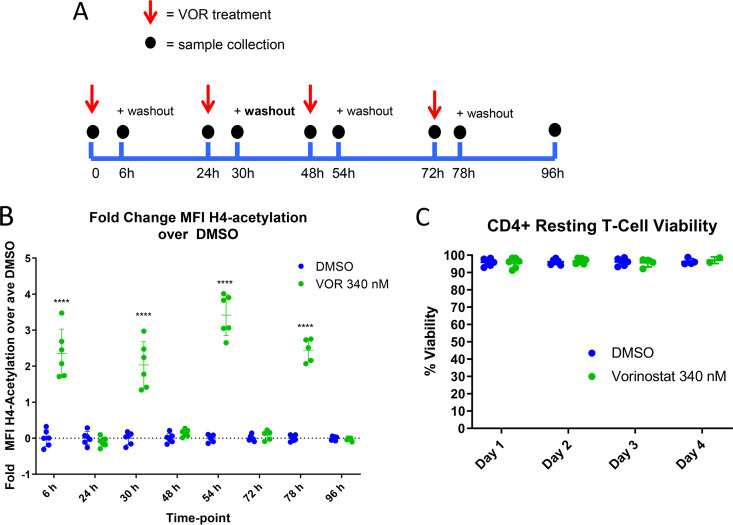
(A) Serial 340 nM VOR exposure in CD4 resting T cells (*n* = 4 donors). (B) Histone acetylation, as measured via flow cytometry, showed a statistically significant (****, *P* ≤ 0.0001) increase at 6 h after each VOR dose, with levels returning to baseline after an 18-h VOR-free period. Error bars represent standard error of the mean. (C) Viability of CD4 T cells throughout the study remained high.

In addition to confirmation of HDACi target engagement and downstream impact on host gene transcription, host gene biomarkers may provide important insights into the HIV latency reversal activity of VOR. Previous clinical studies have demonstrated that induction of HIV ca-RNA in pools of resting CD4 T cells is reduced with repeated q24h doses relative to the induction observed after the first dose (15, 20). To understand whether this phenomenon also occurs at the host gene level, we employed the five validated VOR-responsive genes from the RNA-Seq study (H1F0, IRGM, WIPI49, PHF15, and PRDM10) in tandem with a targeted transcriptome RNA sequencing platform to assess host gene responses with simulated serial q24h VOR dosing in resting CD4 T cells. In these experiments, primary resting CD4 T cells were treated with 340 nM VOR for 6 h each day for 4 days. After each 6-h treatment, cells were washed and incubated with fresh media for 18 h. Histone acetylation and the VOR host gene biomarkers were quantitated at baseline (prior to the VOR treatment) and at 6 h (after VOR treatment) every day over the course of the experiment (Fig. 3A).

**FIG 4:**
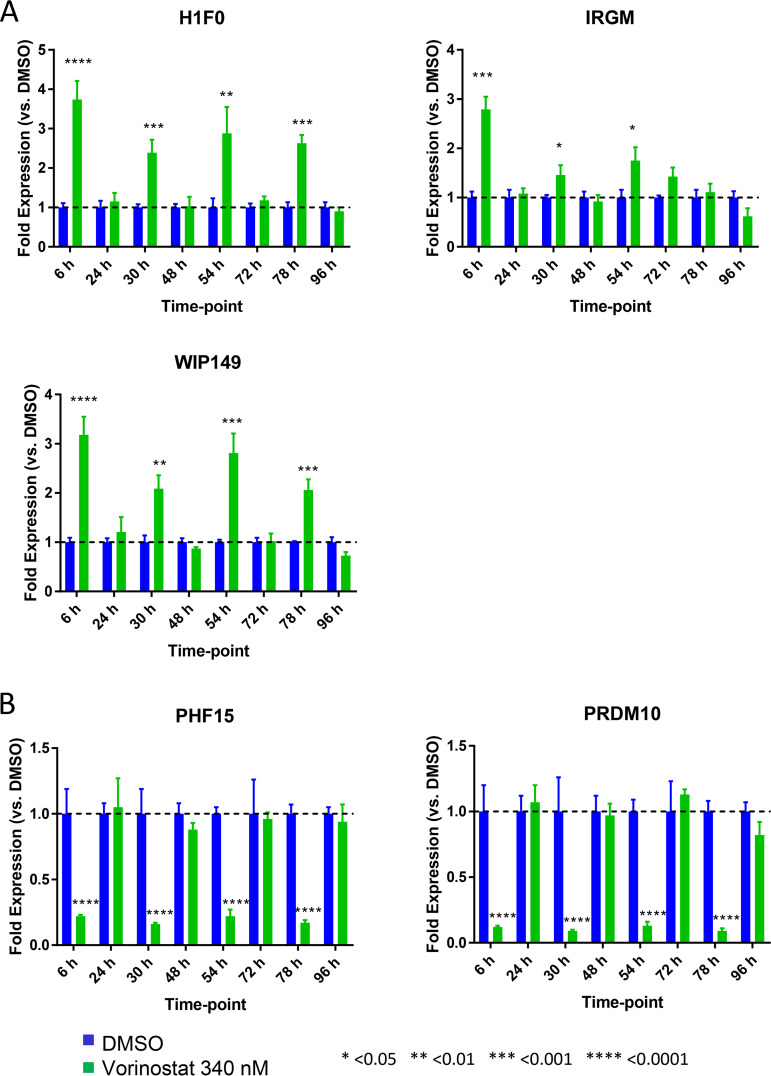
Serial 340 nM VOR exposure in resting CD4 T-cells (*n* = 4 donors) shows statistically significant (one-way analysis of variance [ANOVA]) (A) upregulation or (B) downregulation of five HDACi host gene biomarkers at 6 h post each VOR treatment and a return to baseline expression after an 18-h VOR-free period. Error bars represent standard error of the mean.

We first assessed changes in histone acetylation to provide an epigenetic context for our analysis of host gene modulation after serial HDACi exposure. Samples were gated on the single-cell/live/CD45^+^/CD3^+^/CD4^+^ population, and the fold change in H4 mean fluorescent intensity (MFI) of VOR/DMSO was plotted. Daily, repeated 6-h pulsing of cells with VOR resulted in a robust 2.0- to 3.5-fold increase in histone H4K5/H4K8/H4K12/H4K16 acetylation levels following each VOR pulse, with a return to baseline levels prior to the next stimulation (Fig. 3B). Cell viability was evaluated each day and remained consistently high over the course of the study (Fig. 3C). In addition, the five VOR host gene biomarkers also exhibited a robust change in expression levels following each 6-h incubation with VOR. The increase in expression levels of the three upregulated genes ranged from 2.4 to 3.7, 1.5 to 2.8, and 2.0 to 3.2-fold for H1F0, IRGM, and WIPI49, respectively (Fig. 4A). Likewise, there was an 80 to 90% decrease in PHF15 and PRDM10 expression levels on each day of dosing (Fig. 4B). All VOR host gene biomarkers returned to baseline levels prior to the next pulse of VOR.

**FIG 5:**
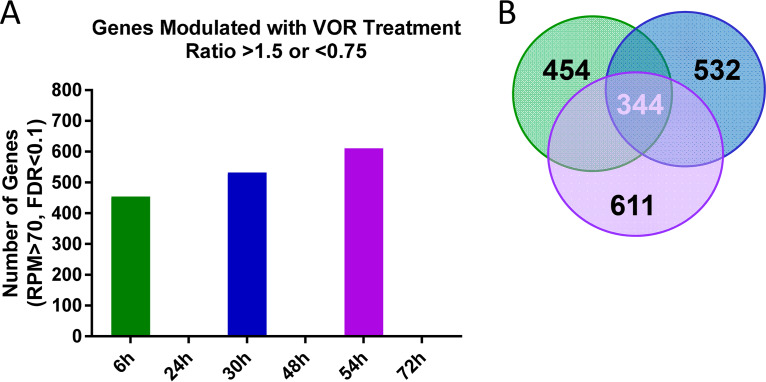
AmpliSeq human transcriptome analysis in resting CD4 T cells evaluating serial 340 nM VOR exposure. (A) Results showed global gene changes 6 h after each VOR treatment, with no expression changes at each 24-h time point after VOR treatment. (B) There were a number of HDACi-responsive genes that were shared across the three VOR stimulations.

Having established that histone acetylation and the five VOR host gene biomarker levels responded robustly after each daily VOR pulse, we sought to examine the host response to repeated VOR stimulations across the whole transcriptome in primary resting CD4 T cells. For these experiments, gene level changes across the transcriptome were evaluated using the AmpliSeq human transcriptome kit, which evaluates 20,000 cellular transcripts. Following daily 6-h VOR treatments, we identified 454 to 611 genes that were modulated by VOR each day, with levels of all genes returning to baseline prior to the next VOR pulse (Fig. 5A and Data Set S2). Of these, 344 genes were consistently modulated after every treatment. This suggests that at least half of the host gene response is conserved with serial VOR dosing (Fig. 5B).

### Serial q24h HDACi exposure *in vitro* in HIV-infected suppressed donor CD4 T cells results in consistent host gene biomarker response.

**FIG 6:**
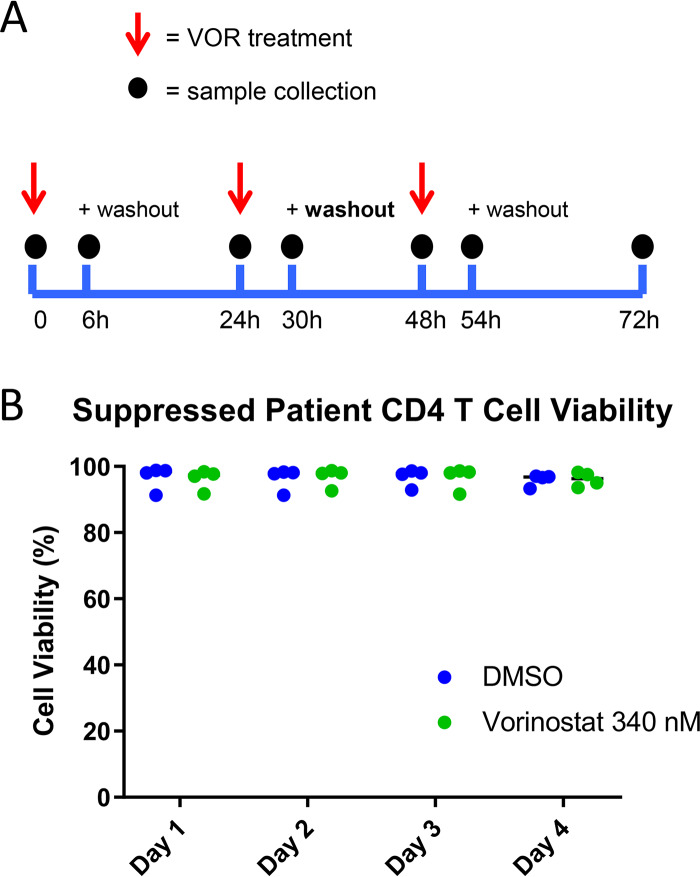
(A) Serial 340 nM VOR exposure in infected suppressed donor CD4 T cells (*n* = 4 donors). Error bars represent standard error of the mean. (B) Cells maintained 80 to 90% cell viability throughout entire study.

**FIG 7:**
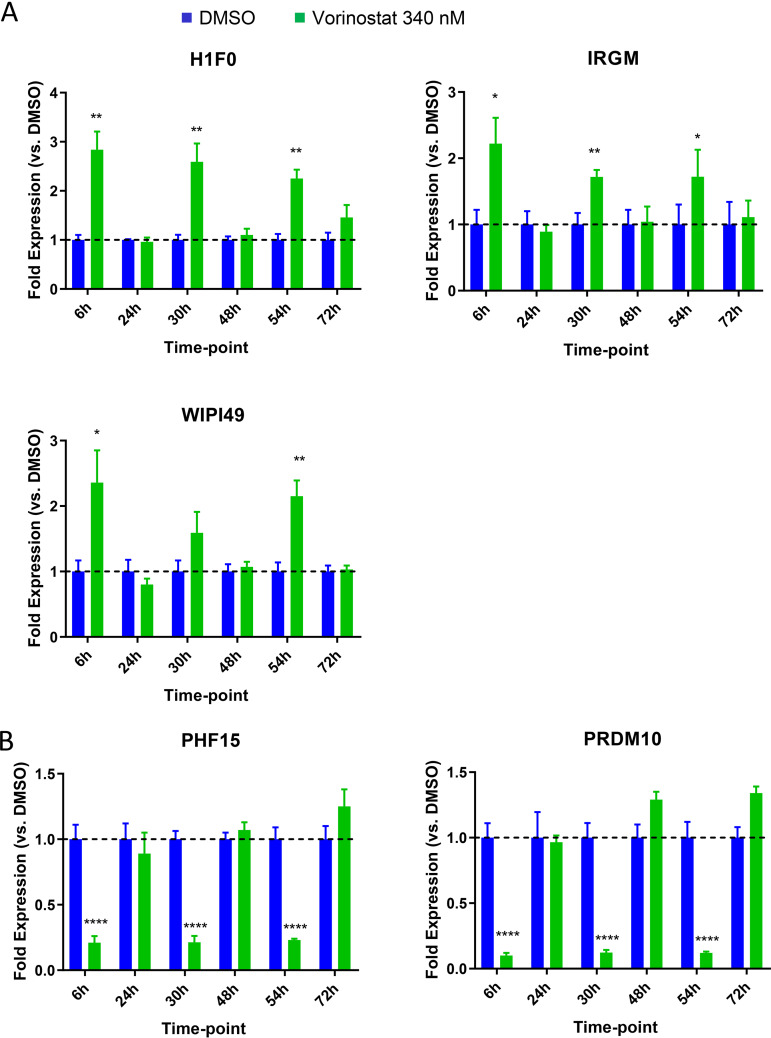
Serial 340 nM VOR exposure in infected suppressed donor CD4 T cells (*n* = 4 donors) shows (A) upregulation or (B) downregulation of five HDACi host gene biomarkers at 6 h post each VOR treatment and a return to baseline expression after an 18-h VOR-free period. Error bars represent standard error of the mean.

We next sought to understand how HIV expression is affected by daily (q24h) VOR dosing in HIV-infected suppressed donor CD4 T cells. Daily treatment with 340 nM VOR was used to stimulate the latent cells for 6 h followed by a washout each day (Fig. 6A). Cell viability throughout the experiment was 80 to 90% (Fig. 6B). The five VOR host gene biomarkers also exhibited a robust change in expression levels following each 6-h exposure to VOR. The increase in expression levels of the three upregulated genes ranged from 2.4 to 3.8, 1.3 to 2.7, and 1.4 to 3.6 for H1F0, IRGM, and WIPI49, respectively (Fig. 7A). Likewise, there was a 68 to 87% and 83 to 91% decrease in expression on each day for dosing for PHF15 and PRDM10, respectively (Fig. 7B). All VOR host gene biomarkers returned to baseline levels prior to the next VOR treatment.

### Host gene biomarker responses in resting CD4 T cells are intact with single and q24h VOR dosing despite reduced HIV ca-RNA induction.

**FIG 8:**
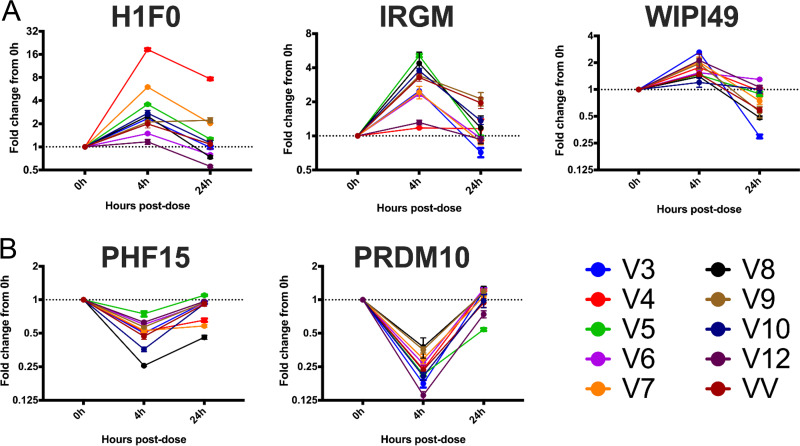
Five HDACi host gene biomarkers demonstrated consistent (A) upregulation or (B) downregulation in PBMC samples from participants who received a single 400-mg dose of vorinostat. Colored lines represent temporal gene expression data from individual participants. Each point is the average of three cDNA replicates, and error bars represent the standard error of the mean. Participant identifiers (IDs) (V) correspond to those in Archin et al. (20). VV represents a participant from a recently completed vorinostat/vaccine study.

**FIG 9:**
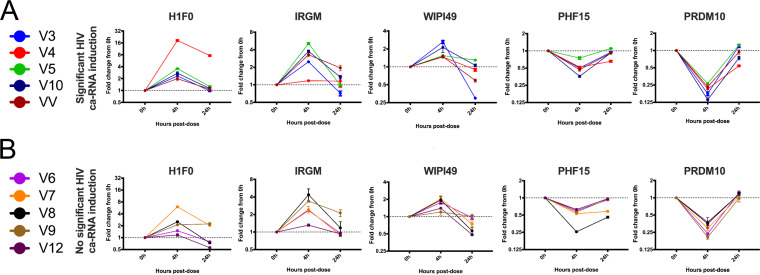
Five HDACi-responsive host gene biomarkers do not predict *in vivo* HIV ca-RNA induction responses. Significant ca-RNA expression (A) versus no significant ca-RNA expression (B). Colored lines represent temporal gene expression data from individual participants. Each point is the average of three cDNA replicates, and error bars represent the standard error of the mean. Participant IDs (V) correspond to those in (20), and “significant ca-RNA induction” indicates statistically significant HIV RNA induction as defined in Archin et al. (20). VV represents a participant from a recently completed vorinostat/vaccine study.

To assess whether the five biomarker genes were modulated after an *in vivo* exposure to VOR, we evaluated the candidate biomarker genes *in vivo* using PBMCs from a recently published study of VOR (20). Samples from HIV-1-infected participants on suppressive cART were collected prior to study start (0 h), 4 h, and 24 h postadministration of a 400-mg dose of VOR. We observed a consistent modulation of host genes 4 h after the dose, which was followed by a return to baseline expression 24 h after the dose (Fig. 8). However, there was no clear difference in host gene biomarker expression in PBMCs for individuals who had a measurable induction of cell-associated HIV RNA in resting CD4 T cells versus those who did not (Fig. 9).

**FIG 10:**
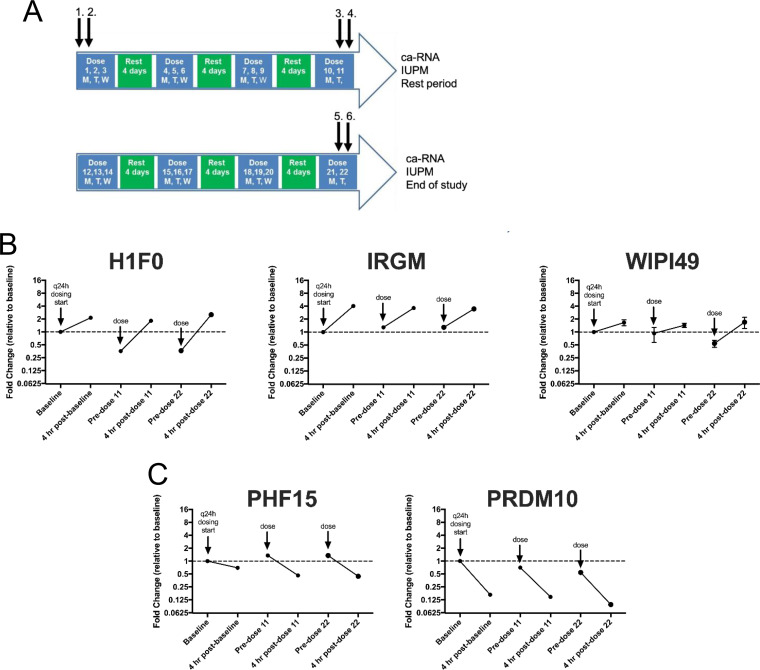
Five HDACi-host gene biomarker responses are intact with multiple doses of vorinostat *in vivo*. (A) Host gene biomarker expression in PBMCs from ART-suppressed, HIV-seropositive participants was assessed before and 4 h after the 1st (1, 2), 11th (3, 4), and 22nd dose (5, 6) of vorinostat on a modified q24h dosing schedule. (B) Upregulated or (C) downregulated host gene biomarkers. Each point is the average of three cDNA replicates, and error bars represent the standard error of the mean. All fold change values are normalized to the baseline sample prior to the start of the multiple-dose regimen. The data plotted are from participant 8 in Archin et al. (15) and are representative of three participants.

A second study (15) assessed HIV ca-RNA in resting CD4 T cells after several cycles of 3 q24h doses of VOR followed by 4 days of rest (Fig. 10A). This multiple q24h dosing resulted in minimal increases in HIV ca-RNA compared to the responses observed at the beginning of the study after a single dose of VOR. Using the validated host gene biomarkers, we sought to understand the host gene response in resting CD4 T cells from these participants after multiple q24h doses of VOR. There was consistent induction and reset of the five candidate host gene biomarkers across multiple q24h doses despite a loss of induction of HIV cell-associated RNA in the resting CD4 T cells relative to a previous single dose of VOR (Fig. 10B and C). This suggests that the reduced HIV transcriptional response with q24h dosing of VOR *in vivo* is due to a cellular refractory state rather than to a loss of transcriptional response to VOR, at least at the level of the host genes examined.

## DISCUSSION

HDACi elicit changes in chromatin structure by preventing the deacetylation of histone marks, resulting in chromatin decondensation and gene transcription. Although treatment with HDACi can elicit HIV mRNA expression in total and resting CD4 T cells in HIV-1-infected participants (14–18), daily serial HDACi exposure led to decreased HIV latency reversal (15). Therefore, we sought to determine the responsiveness of both uninfected and HIV-1-infected cells to serial HDACi exposures.

To this end, we first quantified host cell mRNA expression and histone acetylation levels following exposure to physiological concentrations of VOR in Jurkat cells, CD4 T cells, and HCT116 cells, a colorectal cancer cell line used in the development of VOR. Significant changes in histone acetylation and host gene expression levels were observed during the VOR dosing period; these returned to baseline levels by 24 h. Of these host genes that exhibited altered expression patterns in response to VOR, 1,535 were shared across all three cell types, indicative of a common cellular response. This is consistent with a previous study that found 1,847 differentially expressed genes in primary CD4 T cells that were exposed to 340 nM VOR for 24 h (21).

Previous studies to discover proximal biomarkers of HDACi treatment identified several candidate genes that were consistently modified by VOR in PBMCs (22) or in tumor xenografts (data not shown; Merck & Co., Inc.). These studies, in tandem with our RNA-Seq data for HCT116, Jurkat, and CD4 T cells, identified five candidate VOR-proximal host cell RNA biomarkers (H1F0, IRGM, WIPI149, PHF15, and PRDM10). Using these biomarkers, we sought to determine the responsiveness of cells to repeated exposure to VOR over 4 days *in vitro*. Histone acetylation levels increased during the VOR dosing period and returned to baseline prior to the next dose, providing an internal validation of VOR bioactivity. Measurements of all five candidate biomarkers revealed significant changes with no significant difference in magnitude for 4/5 of the genes on each day 6 h after treatment, returning to baseline prior to the next dose. Although the magnitude of IRGM response is more variable than those of other genes (Fig. 4), we have seen that all five genes remain strongly induced with other HDAC inhibitors (data not shown), as well as across multiple species (data not shown). Furthermore, evaluation of IRGM *in vivo* (Fig. 10) demonstrates consistent induction with sequential VOR exposures. Similarly, histone acetylation levels increased during the VOR dosing period and also returned to baseline prior to the next dose. Collectively, the results from these *in vitro* experiments indicated that cells remained susceptible to daily VOR treatment and exhibited no decline in responsiveness.

Having established that both histone acetylation and host cellular RNA biomarkers were responsive to daily VOR treatment in uninfected cells, we next sought to determine VOR pharmacodynamic activity *in vitro* in treated HIV-positive suppressed donor T cells, as well as *in vivo* in ART-suppressed participants who received single or multiple doses of VOR. In these experiments, CD4 T cells from HIV-infected suppressed donors were treated with VOR for 6 h, followed by an 18-h washout prior to the next exposure. As observed in uninfected CD4 T cells, daily VOR treatment resulted in consistent changes in host RNA biomarker levels.

In a clinical setting, when given a single 400-mg dose of VOR, there is variable induction of HIV ca-RNA in resting CD4 T cells across participants from two published studies (15, 20). Having established five host cell RNA biomarkers of VOR activity, we sought to determine whether this variability in VOR latency reversal activity was a result of differences in cellular responses to VOR. To this end, we compared the induction of our host mRNA biomarkers across participants who had a statistically significant induction of HIV ca-RNA 4 h postdose with that in those who did not. Interestingly, despite differences in induction of HIV ca-RNA across the samples tested, we did not observe a clear difference in host mRNA biomarker response across the participants that demonstrated a statistically significant induction of HIV ca-RNA compared to that in those who did not. VOR appeared to efficiently impact host cell gene expression in all participants at 4 h, followed by a return to the basal state at 24 h. This is consistent with transcriptomic data obtained from whole-blood samples, where a large number of host genes were modulated 2 h after a 400-mg dose of VOR, with a return to the basal state at 8 h and 24 h postdose (16). This study also reported similar gene expression patterns after the 1st and 7th daily doses of VOR, consistent with our findings.

We further evaluated the VOR biomarker genes in PBMC samples from participants who had received multiple q24h doses of VOR. The robust induction of a similar host VOR biomarker gene profile by multiple q24h doses of VOR suggests that the lack of ca-RNA induction in resting CD4 T cells at the same dosing frequency is not due to a lack of cellular responsiveness to HDACi, but rather that proviral response to HDACi is more complex than simply a phenomenon associated with the acetylation of local proviral chromatin.

Several models, which are not mutually exclusive, could explain the complexity of proviral response. Host proteins other than histones might be acetylated, making them unavailable due to degradation or modulation of protein-protein interaction to participate in proviral expression within the next 24-h period. One host transcriptional cofactor that may play a key role in HDACi-mediated latency reversal is p-TEFb (13). In a primary resting CD4 T-cell model, increased p-TEFb was required prior to significant HIV reactivation with VOR. Moreover, p-TEFb release from 7SK small nuclear ribonucleoprotein (snRNP) correlated with HIV reactivation, more so than histone H3 or tubulin acetylation, in cell lines stimulated with HDACi (13).

Alternatively, q24h dosing with VOR could induce factor(s) that antagonize HIV gene transcription, and these effects might resolve 72 h after VOR treatment. Indeed, VOR is known to induce host protein expression of both positive and negative regulators of HIV latency (23). Beliakova-Bethell and colleagues also recently reported a comparison between VOR and romidepsin transcriptional profiles in CD4 T cells and identified two genes that were consistently modulated by both HDACi examined (*SMARCB1* and *PARP1*). Notably, HDACi modulation of these genes is thought to inhibit HIV latency reversal (24). Thus, further investigation of VOR-regulated genes that impact latency is warranted.

Interestingly, in total CD4 cells, Lewin and colleagues observed a correlation between specific gene expression profiles and the levels of total unspliced HIV RNA after VOR exposure (16). However, it remains to be determined whether this relationship is causal because no adjustment was made for baseline HIV ca-RNA expression across participants in the analysis. Therefore, the correlation observed may be due to natural variation in baseline ca-RNA across donors. Future work evaluating the relationship between the heterogeneity of *in vivo* transcriptome responses to VOR and latency reversal is warranted.

Taken together, across *in vitro* and *in vivo* samples, we found that HDACi-inducible gene expression “responds and resets” to a VOR exposure within 24 h, even after multiple q24h doses. The fact that host gene responses remain intact with multiple q24h doses suggests that diminished HIV latency reversal in resting CD4 T cells after HDACi is due to (i) HIV transcription-limiting cofactor availability or (ii) gene modulation favoring HIV latency, rather than related to an absent host gene response to VOR. We speculate that the first dose of an HDACi could reactivate HIV in resting cells with sufficient levels of p-TEFb for release. However, it may be the case that many resting cells do not have sufficient levels of p-TEFb to continue HIV transcription after the first dose and require time to produce more p-TEFb in order to achieve continued reactivation, hence the reduction of HIV ca-RNA in resting CD4 T cells after multiple q24h doses. The low levels of p-TEFb found in resting CD4 T cells, as well as the more efficient HIV reactivation with q72h dosing, supports this notion (25).

Overall, this work identifies and validates five host genes that can serve as robust biomarkers of HDACi and VOR-mediated transcriptional activity and provides insight into the host transcriptional response to repeated doses of VOR in the context of HIV transcription. Importantly, these data suggest that the host transcriptional response to VOR can “respond and reset,” even with q24h dosing. Notably, the transcriptomic profile induced across VOR exposures was similar. This implies that the reduction of HIV transcriptional responses in resting CD4 T cells with q24h dosing, but not with q72h dosing, is likely not due to a refractory gene expression response to VOR but instead to other cell intrinsic HIV transcription-limiting factors, such as availability of P-TEFb. The role of P-TEFb and other transcriptional cofactors in response to VOR in resting CD4 T cells from aviremic donors is an important area of future investigation to inform the use of VOR as an LRA in clinical studies to deplete the latent reservoir.

## MATERIALS AND METHODS

### Cell lines and resting CD4 T cell isolation.

A Jurkat T-cell line containing a mixed population of latent HIV clones (here referred to as Jurkat T cells) was obtained from Jonathan Karn (Case Western Reserve University, Cleveland, OH) and cultured in RPMI 1640 media with 5% fetal bovine serum (FBS), 100 U/ml penicillin (Pen)-streptomycin (Strep), and 2 mM l-glutamine (Thermo Fisher, Carlsbad, CA). HCT116 cells were obtained from the American Type Culture Collection (ATCC, Manassas, VA) and cultured in McCoy’s 5a medium modified with 10% fetal bovine serum (FBS) and 100 U/ml Pen-Strep. Primary resting CD4 T cells were isolated from PBMCs from four healthy donors (Biological Specialty Corporation, Colmar, PA). Resting CD4 T cells were isolated in 2% FBS in phosphate-buffered saline (PBS) by negative magnetic selection using an EasySep custom kit (Stemcell Technologies, Cambridge, MA) containing the following antibodies: anti-CD8, anti-CD14, anti-CD16, anti-CD19, anti-CD56, anti-CD25, anti-CD41, and anti-HLA-DR, plus glyphorin A. After isolation, CD4 T cells were cultured in RPMI 1640 media with 10% FBS and 100 U/ml Pen-Strep at 5 × 10^6^ cells/ml.

### VOR treatment (*in vitro*, single-dose simulation).

Jurkat, HCT116, or primary CD4 T cells were seeded in 6-well culture dishes at different densities so that 1 × 10^6^ cells (Jurkat and HCT116) or 1.5 × 10^7^cells (CD4 T cells) could be harvested at the following time points: 0 h, 6 h, 24 h, 48 h, 72 h, 96 h, and 7 days. Cells were seeded in duplicate for each time point (Jurkat and HCT116) or in separate plates for each of four donors (CD4 T cells) and incubated overnight at 37°C and 5% CO_2_. Immediately prior to dosing with VOR, a 0-h sample of cells was removed and processed for baseline histone acetylation and RNA-Seq gene expression analysis. In brief, the cells were harvested by centrifugation at 800 × *g* for 5 min and resuspended in 1 ml Dulbecco’s phosphate-buffered saline (without calcium chloride or magnesium chloride) (DPBS). 200 μl of cells were removed for enzyme-limited immunosorbent assay (ELISA) histone acetylation analysis (see below), and the remaining 800 μl of cells was pelleted by centrifugation at 1,000 × *g* for 10 min for RNA-Seq and gene expression analysis. All remaining wells were treated with 340 nM VOR (0.1% DMSO [vol/vol]) or vehicle (0.1% DMSO [vol/vol]) in assay medium. After 6 h, a sample of cells was harvested and processed for histone acetylation and RNA-Seq gene expression analysis as described above. For the remaining wells, the cells were washed once with DPBS, resuspended in fresh medium, replated in 6-well dishes, and incubated at 37°C and 5% CO_2_. Cells were harvested at the following time points for histone acetylation and RNA-Seq analysis as described above: 24 h, 48 h, 72 h, 96 h, and 7 days.

### VOR treatment (*in vitro*, serial q24h dose simulation).

Primary resting CD4 T cells from four healthy donors were isolated and cultured (as described above) in two T25 flasks (one each for VOR and a vehicle control) at a density of 5 × 10^6^ cells/ml and incubated overnight at 37°C and 5% CO_2._ For repeat dosing experiments, cells were pulsed with 340 nM VOR (0.1% DMSO [vol/vol]) or vehicle (0.1% DMSO) for 6 h every day for a total of 4 days. After each 6-h VOR pulse period, cells were washed once with DPBS to remove the VOR or vehicle control, resuspended in fresh medium in new T25 flasks, and incubated at 37°C and 5% CO_2_. Cells were sampled from each flask prior to each VOR or vehicle pulse at 0 h (baseline), 24 h, 48 h, and 72 h, as well as after the 6-h VOR/vehicle exposure period at 6 h, 30 h, 54 h, and 78 h. A final sample was taken at the completion of the experiment at 96 h. At each time point listed above, 3 ml of cells was from the T25 flasks from each donor, harvested by centrifugation at 800 × *g* for 5 min and resuspended in DPBS. A 200-μl aliquot was removed for flow cytometry-based histone acetylation analysis (see below). The remaining 800 μl of cells was harvested by centrifugation at 1,000 × *g* for 10 min for AmpliSeq analysis.

### VOR treatment (*ex vivo*, serial q24h dose simulation).

Primary resting CD4 T cells from four HIV-infected suppressed donors, provided by Precision for Medicine, were isolated and cultured (as described above) in two T25 flasks (one each for VOR and a vehicle control) at a density of 3 × 10^6^ cells/ml and incubated overnight at 37°C and 5% CO_2._ For repeat dosing experiments, cells were pulsed with 340 nM VOR (0.1% DMSO [vol/vol]) or vehicle (0.1% DMSO) for 6 h every day for a total of 3 days. After each 6-h VOR pulse period, cells were washed once with DPBS to remove the VOR or vehicle control, resuspended in fresh medium in new T25 flasks, and incubated at 37°C and 5% CO_2_. Cells were sampled from each flask prior to each VOR or vehicle pulse at 0 h (baseline), 24 h, and 48 h, as well as after the 6-h VOR/vehicle exposure period at 6 h, 30 h, and 54 h. A final sample was taken at the completion of the experiment at 72 h. At each time point listed above, 2 ml of cells was removed from the T25 flasks from each donor, harvested by centrifugation at 800 × *g* for 5 min, washed with DPBS, and harvested by centrifugation at 1,000 × *g* for 10 min for gene expression analysis.

### Histone acetylation ELISA.

Frozen cell pellets from the VOR *in vitro* simulation studies (see above) were analyzed by a histone acetylation ELISA described previously (26) with the following modifications: cell pellets were lysed in 220 μl 1% Triton X-100/PBS and incubated at room temperature for 20 min prior to being added to the coated and blocked ELISA plate; plates were incubated for 10 min after Tropix CDP-Star Sapphire II substrate addition.

### Histone acetylation flow cytometry.

Cell samples from each time point from the VOR *in vitro* single- or multiple q24h-dose simulation studies (see above) were washed with DPBS and stained with the fixable violet dead cell stain kit (Invitrogen, Carlsbad, CA) for 20 min. Cells were then stained with an extracellular antibody cocktail in fluorescence-activated cell sorting (FACS) buffer (PBS + 5% FBS) containing anti-CD3-fluorescein isothiocyanate (FITC) clone 1F4, anti-CD4-PE clone W3/25, and CD45-PeCy7 clone OX-1 (BioLegend, San Diego, CA) for 30 min at room temperature. Cells were fixed with 4% paraformaldehyde and kept at 4°C until the last time point was collected. Fixed samples were washed and permeabilized using the eBioscience Foxp3/transcription factor staining buffer set (Invitrogen, Carlsbad, CA) and stained with the anti-H4 K5/8/12/16 antibody clone 3HH4-4C10 (Millipore, Burlington, MA) conjugated to AF647 for 30 min at room temperature. Samples were analyzed on the LSR II (BD Biosciences, San Jose, CA) and in FlowJo (version 10).

### RNA-Seq analysis.

Total RNA was isolated from the *ex vivo* single-dose simulation study cell pellets (described above) using the RNeasy minikit (Qiagen, Valencia, CA) according to the manufacturer’s protocol with the following modifications. Samples were homogenized using 600 μl RLT lysis buffer (supplied with kit) plus β-mercaptoethanol and 5 mm stainless steel beads in a TissueLyser instrument (Qiagen, Valencia, CA); RNA was eluted with 30 μl RNase-free water. RNA samples were quantitated, and the RNA integrity number (RIN) was determined using the RNA 6000 Nano (Jurkat and HCT116) or RNA 6000 Pico (CD4) kits and the 2100 Bioanalyzer instrument (Agilent, Santa Clara, CA) according to the manufacturer’s protocol. RNA (1 μg) for each sample was sent to Quintiles-Quest Expression Analysis, Inc. (Morrisville, NC) for RNA-Seq analysis. RNA samples were converted into cDNA libraries using the Illumina TruSeq stranded total RNA sample preparation kit (catalog no. RS-122-2303; Illumina). Final libraries were quantified via qPCR, normalized to 2 nM, pooled, and sequenced on the Illumina platform. Sequences from the RNA-Seq analysis were analyzed using DEseq2 software to evaluate genes modulated with fragments per kilobase per million (FPKM) of >50 and a false-discovery rate (FDR) of <0.1 for each time point.

### AmpliSeq human transcriptome analysis.

Total RNA was isolated from the VOR *in vitro* q24h multidose simulation study cell pellets (described above) using the RNeasy minikit (Qiagen, Valencia, CA) according to the manufacturer’s protocol with modifications described above. RNA samples were quantitated using the Qubit RNA high-sensitivity (HS) assay kit and the Qubit 3.0 fluorometer (Life Technologies, Carlsbad, CA). RNA (30 ng) was converted to cDNA using the SuperScript Vilo kit (Life Technologies, Carlsbad, CA) per the manufacturer’s protocol. Library barcoding, preparation, and sequencing were carried out per the manufacturer’s protocols using the following Ion Torrent kits on the Ion Chef Prep station and the Ion Torrent S5-XL (Life Technologies, Carlsbad, CA): Ion AmpliSeq transcriptome human gene expression panel Chef-ready kit (catalog no. A31446), Ion 540 chip kit (catalog no. A27766) and Ion 540 Kit-Chef (catalog no. A30011) (Life Technologies, Carlsbad, CA). Eight samples per 540 chip were sequenced with 8 to 10 million reads/sample. Sequencing from the AmpliSeq analysis was analyzed using Partek Flow software to evaluate genes modulated with RPM of >70, an FDR of <0.1, and a VOR/DMSO ratio of >1.5 or <0.75 for each time point.

### Gene expression analysis (*in vitro* studies).

HDACi-modulated genes identified in previous studies and in the transcriptome analyses were also evaluated in RNA samples from *in vitro* VOR simulation studies. RNA previously isolated for each study (see above) was converted to cDNA with the high-capacity cDNA reverse transcription kit (Life Technologies, Carlsbad, CA) per the manufacturer’s protocol at a concentration of 20 ng/μl. Twelve HDAC-modulated genes (Table 1) and Human GAPD (GAPDH) endogenous control (catalog no. 4310884E) from Life Technologies (Carlsbad, CA) were evaluated in samples from *in vitro* VOR studies using the TaqMan Fast advanced master mix and analyzed according to the manufacturer’s instructions on the QuantStudio 12K Flex instrument (Life Technologies, Carlsbad, CA). Changes in gene expression for VOR-treated samples were compared to gene expression in DMSO controls at the same time point using the threshold cycle (2^−ΔΔ^*^CT^*) method (27).

### Gene expression analysis (*in vivo* studies).

PBMCs were collected and viably cryopreserved at the indicated time points postdose(s). Cells were thawed and immediately pelleted for RNA extraction using the Qiagen RNeasy kit according to the manufacturer’s instructions. RNA was quantified using a NanoDrop 1000 spectrophotometer (Thermo Scientific). Three reactions of cDNA per sample, each with an RNA input of 500 ng, were prepared using the Maxima cDNA synthesis kit with dsDNase according to the manufacturer’s instructions (Thermo Scientific). cDNA reactions were diluted 1:4 and subsequently used for qPCR in technical duplicates. Twenty-microliter qPCR reaction mixtures consisted of 10 μl of QuantiTect multiplex PCR NoROX mastermix (Qiagen), 0.25 μl of AmpErase uracil *N*-glycosylase (Thermo Scientific), 2 μL of diluted cDNA, 400 nM or 1× primers, and 200 nM or 1× probes. Primer/probe sets for assessment of HDACi-modulated gene expression included previously validated primer/probe sets for H1F0, IRGM, and RPL27 from Integrated DNA Technologies (22) or predesigned 20× TaqMan assays for PHF15 (Hs00959516) and PRDM10 (Hs00999748) from Thermo Fisher Scientific (Waltham, MA). qPCR cycling was conducted on the Bio-Rad C1000 Touch thermal cycler as follows: 50°C × 2 min and 95°C × 15 min, followed by 40 cycles of 94°C × 1 min and 64°C × 1 min. Cycle quantification (*C_q_*) values were determined using the automatic threshold analysis in Bio-Rad CFX Maestro software version 1.1. Expression of HDACi-modulated host genes was assessed using the 2^−ΔΔ^*^CT^* method (27). RPL27, a gene known to be nonresponsive to HDACi stimulation, was used as a reference gene (21).

## Supplementary Material

Supplemental file 1
